# Post-traumatic stress disorder following childbirth: prevalence and associated factors—a prospective cohort study

**DOI:** 10.1007/s00404-022-06460-0

**Published:** 2022-03-01

**Authors:** J. Steetskamp, L. Treiber, A. Roedel, V. Thimmel, A. Hasenburg, C. Skala

**Affiliations:** Department of Gynecology and Obstetrics, Mainz University Medical Center, Langenbeckstr. 1, 55131 Mainz, Germany

**Keywords:** Post-traumatic stress disorder, Childbirth, Labor, Mode of delivery, Vulnerability

## Abstract

**Objective:**

Traumatic experiences during or after childbirth are subject of intense discussions in mainstream and social media as well as in scientific literature. Aim of this evaluation is to estimate the prevalence of post-traumatic stress disorder (PTSD) following childbirth in postpartum women and to evaluate the influence of maternal, obstetrical and neonatal characteristics on the degree of PTSD symptoms measured by the Impact of Events Scale questionnaire (IES-R).

**Methods:**

In total, 589 women who gave birth in the University Medical Center Mainz, Germany in 2016, participated in a survey within the first days after birth. Of these, 278 also participated 6 months later. All participants received the validated Impact of Events Scale questionnaire (IES-R). The influence of maternal, obstetric and fetal parameters on PTSD score was evaluated.

**Results:**

PTSD overall prevalence was 2.9%. Patients with PTSD had significantly less often personal support during labor (*p* < 0.001). Maternal age (*p* < 0.001), parity (*p* < 0.001), migration background (*p* < 0.001), mode of delivery (*p* < 0.001) and assistance during labor (*p* < 0.001) were parameters significantly influential on the PTSD symptom level measured by the IES-R.

**Conclusions:**

Maternal PTSD prevalence after childbirth seems to be quite rare with 2.9%. Nevertheless, recent findings assume that this prevalence may only represent the “tip of the iceberg”. PTSD after childbirth should not be underestimated. As PTSD depends on personal vulnerability and existing risk factors, patients at risk have to be detected before childbirth, which appears to be challenging especially for obstetric and family care professionals.

## Introduction

Childbirth is a life-changing experience with a profound effect on almost every aspect of a women’s physical, mental and social life. Whereas a vast majority succeeds in adapting to this new situation in life, a considerable proportion of all mothers develop psychiatric disorders [1–5]. Childbirth is generally seen as one of the major events in parents’ life and it is related to intense positive emotions. However, bearing a child is a borderline experience in women’s life. During labor, the mother-to-be is at the mercy of this special situation, due to the nature of childbirth, including the experience of pain and sometimes the first confrontation with hospitalization and medical intervention. Different groups are promoting childbirth in different ways. Therefore, the childbirth situation may be overcharged with unrealistic or unreachable expectations. Many deliveries are different from parents’ own expectations and wishes. In case of complications during labor, a sudden reaction is often required on the part of the obstetric team to ensure the health of both mother and child. In this situation, there is no time to adapt, and the necessary treatment can be completely unexpected, undesired and overwhelming. In this context, the discussion about obstetric violence arises [6, 7]. Traumatic experiences can be the result, which might affect the upcoming months or years [8]. 20 to 30% of all mothers are assumed to experience childbirth as traumatic, while 10% might have a severe traumatic stress response in the puerperium. On the other hand, only 0.8 to 6.9% of all women giving birth are reported to meet full diagnostic criteria of a post-traumatic stress disorder as a consequence of delivery [9].

A global movement against obstetric violence was founded in 2013, called the “roses revolution” [10], organizing on the day against violence towards women in the November of every year. Young mothers who experienced violence during labor are invited to put a red rose in front of the delivery room, where their child was born. A photo of this rose in the obstetric ward should then be posted on Facebook. In 2015, a red rose was deposited at the door to the delivery room of University Medical Center Mainz for the first time.

With the experience of finding a red rose in front of the door of the delivery room, we intended to find out how women do experience labor in the obstetric clinic of University Medical Center Mainz.

## Materials and methods

As a matter of quality management, we initiated the following verification of the public accusation of obstetric violence in delivery rooms. We performed a 1-year prospective longitudinal analysis from January until December 2016. Mothers and their partners were interviewed on the topic of “Birth experience” within the first 6 months after birth.

In 2016, a total of 1989 births took place in the Department of Obstetrics and Women’s Health at the University Medical Center in Mainz. During this period, all postpartum women who delivered on term were consecutively approached at maternity ward in the first few days following delivery and asked to participate in a survey. 1193 women could be contacted after delivery, 989 were interested to participate. Finally, 589 (59.6%) were recruited for the study. We excluded those women whose child had a severe disease, who did not speak German or English, were illiterate and those who declined to give an informed consent. The mothers received the Impact of Events Scale questionnaire (IES-R) within the first days postpartum to assess post-traumatic stress disorder (PTSD). Six months later, another survey took place and the IES-R questionnaire was sent to 589 women, 278 responses returned by mail.

### IES-R

The IES-R is a self-report scale designed to detect PTSD. It has been shown to have satisfying internal consistency, test–retest reliability and concurrent validity. It consists of 22 questions, 7 questions dealing with intrusion, 8 questions with avoidance, and 7 questions with hyperarousal. A score is calculated and lies in between a spread of − 4.36 and 2.99. A score > 0 gives evidence for a PTSD [11].

PTSD scores were put into relation to maternal and obstetric parameters. The maternal parameters were age, parity, migration status, pregnancy-related diseases, the presence of assistance during labor. Obstetric parameters were duration of labor, birth-related injuries including episiotomy, mode of delivery, weight, and head circumference of the child.

## Data analysis

Statistical analysis was performed using SPSS (SPSS 22.0, IBM, Chicago IL; USA).

Descriptive analysis took place using total numbers and percentages for categorial variables while means with standard deviations were given for continuous variables. As normal distribution could not be expected for all groups, group comparison was performed using the nonparametric Mann–Whitney *U* Test. The comparison of the extend of IES-R scores in relation to the mode of delivery contained more than two grouping variables and was, therefore, done by the Kruskal–Wallis Test. Post hoc analysis was performed applying the Dunn–Bonferroni-Correction; statistical significance was computed at p < 0.05. To predict risk factors for PTSD, independent variables were then included into a multilinear regression analysis. Odds ratio and 95% CI were calculated for the associated variables.

All patients gave written consent for participating in this evaluation, which was performed in accordance with the ethical standards of the Johannes Gutenberg University Mainz and with the 1964 Helsinki Declaration and its later amendments or comparable ethical standards.

## Results

**Fig. 1:**
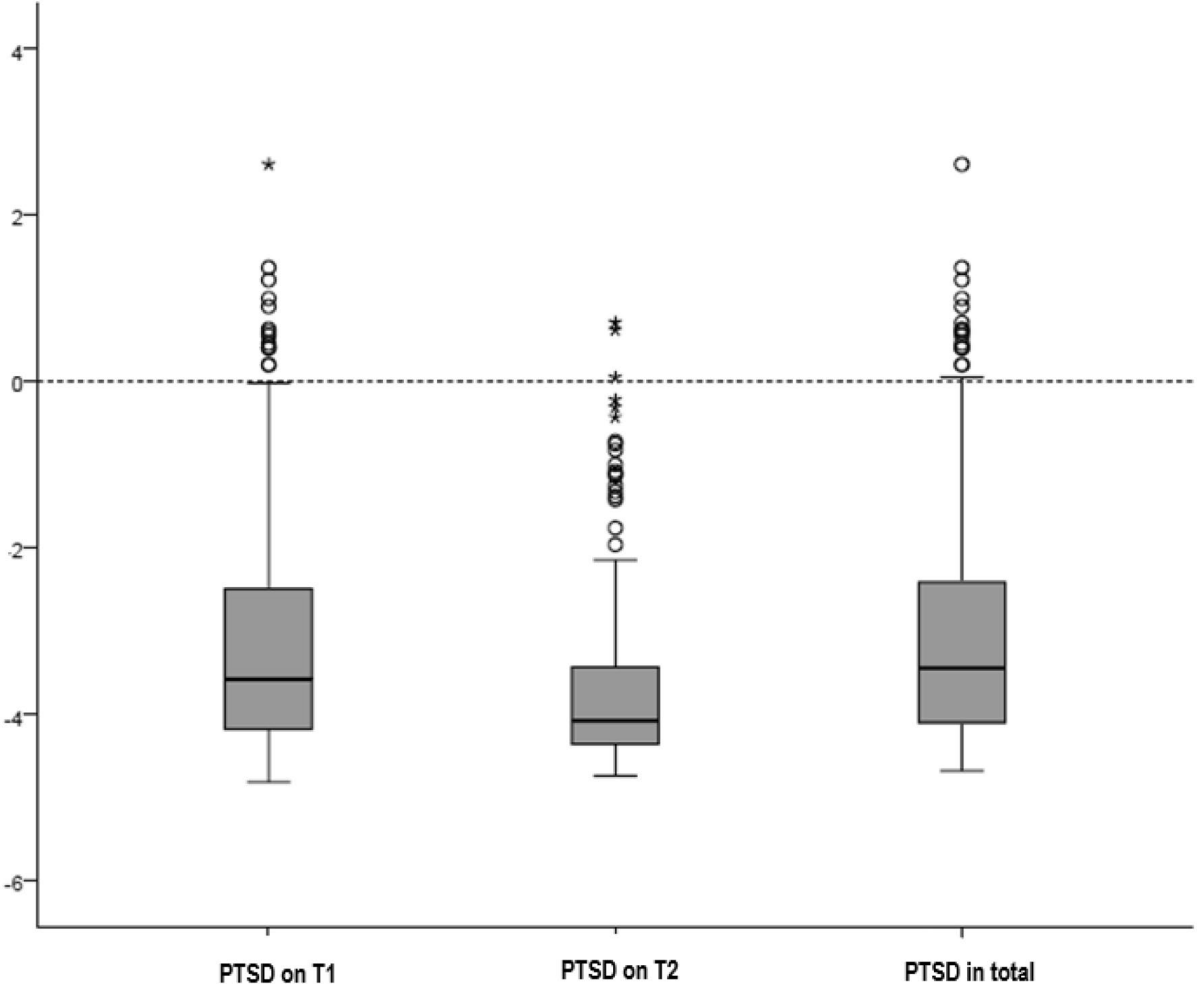
PTSD score on T1 and T2, overall (IES-R, cutoff > 0)

1. Prevalence of PTSD 589 women were evaluated with the Impact of Events Scale questionnaire. 278 patients answered the questions twice. There was no difference in the answers of the two evaluations (Fig. 1). The higher scores were included in the following evaluation.
  In our survey, 2.9 percent (17/589) of all patients met diagnostic criteria for PTSD, all of them having a score above zero.
  In a first step, PTSD patients were compared to those being healthy. In a second step, the influence of different obstetric and maternal parameters on PTSD symptom severity measured by IES-R score expression was evaluated for all patients, independent from the presence of PTSD diagnosis.
2. Comparison of patients with PTSD with those without PTSD
  The comparison of both patient groups is demonstrated in Table 1. Patients with and without PTSD did not differ significantly in age (*p* = 0.112), parity (*p* = 0.682) and gestational age (*p* = 0.33) at labor. Furthermore, the prevalence of pregnancy-related diseases was not different between both groups. PTSD tends to be seen more often in patients with a migrant background (*p* = 0.007). A significant difference can be seen in relation to assistance during labor (*p* < 0.001). Patients who had a companion were less likely to develop PTSD (Table 1). No difference was between the two groups concerning obstetric parameters as follows: duration of labor (*p* = 0.716), babies’ weight (*p* = 0.095) and birth injuries (*p* = 0.623) (Tables 1 and 2).
  Regression analysis was performed (Table 3) to identify independent risk factors for PTSD, showing maternal age and the presence of a companion during labor to be influential on the occurrence of PTSD. Increasing maternal age turned out to lower the risk of PTSD. With every additional year of age, the risk for a PTSD is reduced by 10.2% (RK − 0.107, OR 0.898, *p* = 0.038). If a companion is present at labor, the risk for PTSD is reduced by 84.3% (RK − 1.854, OR 0.157, *p* = 0.001).
3. Influence on PTSD score

**Table 1:** Influence of maternal parameters on PTSD

	Total	PTSD	No PTSD	*p*
*N*	589	17 (2.9%)	572 (97.1%)	
Age	3244 (16–49)	30.12	3251	0.112
Parity	167 (1–5)	165	167	0.682
Gestational age at delivery	3963	3959	39,63	0.33
Migration background				
Yes	129 (21.9%)	7	122	
No	460 (78.1%)	10	450	0.07
Companion during labor				
Yes	383 (65.0%)	4	379	
No	206 (35.0%)	13	193	** < 0.001**
Mode of delivery				
Spontaneous delivery	351 (59.6%)	7	344	0.331
Primary caesarean	100 (17%)	3	97	
Secondary caesarean	103 (17.5%)	5	98	
Vacuum extraction	35 (5.9%)	2	33	
Birth injury	322 (55%)	314	8	0.623

Statistically significant results are shown in bold

**Table 2:** Obstetric parameters in PTSD and non-PTSD women

		Mean	Range	SD	*P*
Duration of labor (minutes)	All	196.33	10–1024	238.718	0.716
	PTSD	213.50	16–820	268.445	
	No PTSD	195.91	1–1024	238.193	
Birthweight	All	3319.92	1440–4900	556.854	0.095
	PTSD	3043.57	1440–3850	623.299	
	No PTSD	3326.65	1470–4900	554.024	
Cranial circumference	All	34.77	27–39	1.575	0.460
	PTSD	34.29	30–36	1.773	
	No PTSD	24.79	27–39	1.570

**Table 3:** Univariate logistic regression probability of occurrence for PTSD

	Regressions’ coefficient	Odds ratio	*P* value
Age	− 0.107	0.898	***0.038***
Parity	− 0.034	0.966	0.911
Hypertension	1.412	4.104	0.074
Gestational diabetes	− 0.449	0.638	0.667
Companion	− 1.854	0.157	***0.001***

Statistically significant results are shown in bold

The influence of maternal, obstetric and fetal factors on the IES-R score is demonstrated in Table 4.

**Table 4:** Maternal, obstetric and fetal parameters in relation to IES-R score

	Total	PTSD score	*p*
Maternal factors			
Age			** < 0.001**
Migration background			** < 0.001**
•Yes	130	− 276	
•No	459	− 318	
Parity			** < 0.001**
•Primipara	295	− 291	
•Second para	217	− 325	
•Multipara	77	− 334	
Pregnancy-related disease			
Obesity			0.440
•Yes	84	− 299	
•No	505	− 310	
Hypertension			0.346
•Yes	21	− 285	
•No	568	− 310	
Gestational diabetes			0.950
•Yes	52	− 313	
•No	537	− 309	
Labor			
Assisting companion			** < 0.001**
•Yes	383	− 324	
•No	206	− 281	
Birth injuries			**0.02**
•Yes	322	− 319	
•No	267	− 296	
Mode of delivery			
•Spontaneous delivery	351	− 327	
•Primary caesarean	100	− 290	
•Secondary caesarean	103	− 270	**0.001**
•Vacuum extraction	35	− 29	

Statistically significant results are shown in bold

Maternal factors appeared to influence PTSD symptom severity. Older patients showed a significantly lower PTSD score (*p* < 0.001), while patients with a migrant background showed higher PTSD scores (*p* < 0.001). Furthermore, parity had an influence on the extent of the IES-R score (*p* < 0.001). On average, patients showed the highest PTSD symptom scores after their first birth. Pregnancy-related diseases such as hypertension (*p* = 0.346), gestational diabetes (*p* = 0.950) or obesity (*p* = 0.440) did not show any effect on the IES-R score.

In addition, labor-related factors had an influence on PTSD symptom severity. Patients receiving assistance during labor had significantly lower scores (*p* < 0.001), patients with birth injuries had significantly higher scores (*p* = 0.02). The mode of delivery showed a clear influence. Patients after spontaneous delivery had the lowest IES-R score (-3.27). The highest IES-R score was seen in the case of a secondary caesarean (-2.70). There was a significant difference between patients after spontaneous delivery and secondary caesarean section (*p* < 0.001). Duration of labor and fetal factors did not show to have an effect on PTSD extent.

Multiple linear regression (Table 5) could affirm the influence of parity (RK − 0.2, *p* = 0.003), maternal age (RK − 0.024, *p* = 0.035), migration background (RK 0.445, *p* < 0.001), mode of delivery (RK 0.103, *p* = 0.035) and assistance during labor (RK − 0.369, *p* = 0.001) on PTSD score.

**Table 5:** Multilinear logistic regression: PTSD score in dependence to anamnestic and obstetric variables

	Regressions’ coefficient RK	*P*
Mode of delivery	0.103	**0.035**
Assisting companion	− 0.369	**0.001**
Migration background	0.445	** < 0.001**
Obesity	0.104	0.472
Hypertension	0.152	0.577
Gestational diabetes	− 0.016	0.928
Birth injury	− 0.032	0.781
Age	− 0.024	**0.035**
Parity	− 0.200	**0.003**
Duration of labor	0.000	0.206
Cranial circumference	0.015	0.644

Statistically significant results are shown in bold

## Discussion

This postnatal survey could fortunately show a particularly low PTSD incidence after childbirth. 589 women were willing to answer the questions of the IES-R questionnaire. Six months later, 278 patients, just 50% felt up to answering the questions another time. There was no significant difference in the quality of answers, but somehow there remains some insecurity concerning the 50% drop out. Over all 17 out of 589 participants developed PTSD. These 17 patients did show significant differences to the rest of the group concerning support during labor by a person in company (*p* < 0.001). Besides the effect of birth, our analysis demonstrated that the maternal age (*p* < 0.001), parity (*p* < 0.001), migrant background (*p* < 0.001), assistance during labor (*p* < 0.001) and the mode of delivery (*p* = 001) do influence PTSD symptom severity. The older the woman, the lower the risk for traumatic experience during labor, as we could show that with every year of age the risk for PTSD is reduced by 10.2%. Mothers showed a higher score after first labor, than after the second or any further childbirths. Patients with migrant background showed significantly higher IES-R scores. Women who had to attend to the obstetrical ward alone during labor showed significantly higher IES-R scores. Assistance during labor by a private person reduces the risk for PTSD by 84.3%. Mode of delivery also had some influence on IES-R scores, but only patients after spontaneous delivery and patients after unexpected caesarean differed significantly from each other. Women undergoing vaginal operative delivery or a planned caesarean did not differ from patients after spontaneous delivery.

Although the numbers in our survey are low, postpartum PTSD is a manifest problem and should not be underestimated, as, in particular, avoidant behavior is one of the cardinal symptoms of PTSD. Avoiding situations and thoughts that might evoke stressful emotions can be the reason for some persons concerned not to reveal the problem. Therefore, the real extend of PTSD tends to be underestimated. We are aware, that the real incidence of patients with PTSD after delivery might be higher, as only 589 participated in this survey and barely 50% were ready to evaluate their experiences a second time 6 months later. Avoidance might be a possible reason why patients did not participate at all or only once. A meta-analysis by Yildiz found a rate of 4% up to 18.5% in high-risk groups [12]. They included 28 studies with 8511 patients, which evaluated the prevalence of PTSD after childbirth. To avoid confounding with acute stress disorder, one key inclusion criteria was the result of a postpartum assessment at least 1 month after birth. Several clinical instruments were used, self-report questionnaires as well as structured diagnostic interviews to estimate the prevalence of PTSD. In their review, Ayers et al. assumed that up to 10% of all women giving birth have a severe traumatic stress response to delivery, but only 1–2% develop chronic PTSD. These patients are supposed to have a higher vulnerability or show other risk factors. The diathesis stress approach [9] proposes that characteristics of an event interact with the individual vulnerability or strength. This determines whether a person develops PTSD or not.

Following Ayers’ model risk factors can be divided into pre-, peri- and postnatal. The main prenatal risk factors are psychosocial factors (as anxiety, poor coping, and low support) and a history of psychiatric problems. Concerning risk factors intrapartum, the mode of delivery, pain in labor and support in labor as well as subjective factors such as violation in expectations and lack of control become important. The postnatal risk factors are additional stress, low support, and possibly complex emotions such as guilt, shame, and blame. Nevertheless, only few young mothers develop PTSD, although the experience of labor and delivery was extreme. The individual vulnerability or strength of a young woman is a personal feature that cannot be easily assessed, not to mention influenced.

A feasible strategy to reduce the risk of developing PTSD should include three key points: awareness of the individual vulnerability of every patient in the delivery room, identification of pre-existing risk factors and avoidance of every additional risk factor.

Identifying possible risk factors is a process which certainly should already be performed as soon as possible ante partum. Our data show that young patients, primiparous women, patients with migration background and patients without support are assumed to have a higher risk. Ayers’ model clearly shows that birth and especially the experience of giving birth does not start in when attending to the labor ward but with the very beginning of the pregnancy. Obstetricians and midwifes who take care of a pregnant women during pregnancy should check if there are prenatal risk factors, such as anxiety, poor coping or low support, if there are financial problems and which sorrows the pregnant women has to deal with. Low-threshold access to any kind of psychosocial as well as economic support of those women at risk should be granted. During pregnancy, the process of delivery should be explained in every step in order to keep couples informed about what they have to expect. In this context, it is necessary to keep parents’ expectation in realistic dimensions. The importance of personal support by a companion during delivery should be explained.

During the first presentation in the delivery room, the mother-to-be and the obstetric professionals can get acquainted to each other, see how they can communicate, present their expectation of delivery. This is a good time to check if the mother-to-be is worried, has support, feels safe, does speak the same language both in a linguistic and a figurative sense. Getting involved with the person from a holistic point of view leads to empathy and creates confidence.

The process of delivery cannot always be influenced in the way that all unexpected events or dramatic situation can be avoided. A switch from the desired spontaneous delivery to an unexpected caesarean section, or even an emergency caesarean, is often unpreventable. It is important that young parents have confidence in their obstetric team. For obstetric professionals, dealing with women giving birth to a child might be a daily routine. For the mother-to-be, giving birth is a borderline situation. As the perception of the same situation is so different, this contrast can result in a lack of comprehension towards the women’s feelings. Considering the relation of both partners, women in labor are dependent and certainly in a weaker position. Nevertheless, awareness of this difference in perception is the key to creating a setting in which women giving birth can feel safe and self-confident. First, obstetric professionals have to recognize that a delivery might be experienced as extremely stressful. The individuality and vulnerability of each woman in the delivery room should always be taken into consideration. It is important to recognize every mother-to-be as an individuum, with particular senses, personal reactions and individual emotions, not just a delivery. There are mothers-to-be who need more attention, more care, more support. It is important to recognize hints and gestures. This way of empathic behavior in advance paves the way for young parents to trust their obstetric team, letting the experience of violence and trauma during labor occur less often. In many cases, there are possibilities to reduce risks and solve problems before traumatic experiences arise.

We are aware of the limitations of this study. Maternal PTSD prevalence after childbirth seems to be quite rare with 2.9%, which probably only represents “tip of the iceberg”. The real incidence of PTSD after delivery turns out to be hardly detectable. First, 400 out of 989 women who were initially interested did not participate in this survey after all. Second, there is a drop out of nearly 50% after the first investigation. Women have lots of reasons after delivery not to participate as a new part of family life begins. But we cannot be sure that one reason not to participate is PTSD itself.

Besides that, the initial investigation within the first 3 days after delivery seems to be very prompt. Acute stress disorders might not be distinguishable from PTSD. From a practical point of view, this early approach was necessary to inform patients and to get written consent as a condition for the second approach.

The roses revolution and the first rose in front of the delivery room has made us aware of an issue which for long was not the center of our attention. With our survey, we wanted to have an impression of the severity of this problem. Our results lead us to the conclusion that this issue needs to be addressed by a multicentered approach and for a more extended period in the life of young mothers.

## Implications for practice

The experience of childbirth occurs to be highly individual and influenced by a variety of intrapersonal as well as extrinsic factors. Traumatic childbirth experience is influenced by the mother’s personal vulnerability and coinciding risk factors. As the mothers’ vulnerability turns out to be strongly connected to her personality, it might be barely possible for obstetric professionals to have an influence on the individual vulnerability itself. Therefore, the identification and elimination of possible risk factors moves into the center of attention. When obstetric professionals succeed to identify and to minimalize these risk factors as early as possible and to provide confidence, the chance of avoiding PTSD becomes realistic even in vulnerable patients.

## Data Availability

The data are available.

## Abbreviations

IES-R: Impact of Events Scale questionnaire revised
PTSD: Post-traumatic stress disorder
